# Supplementary material for: Evaluation of calcium-sensitive adenylyl cyclase AC1 and AC8 mRNA expression in the anterior cingulate cortex of mice with spared nerve injury neuropathy
Source: Neurobiol Pain. 2021 Dec 21;11:100081. doi: 10.1016/j.ynpai.2021.100081 (PMC8715370; doi:10.1016/j.ynpai.2021.100081)
Supplement: Supplementary data 1 [file mmc1.docx]

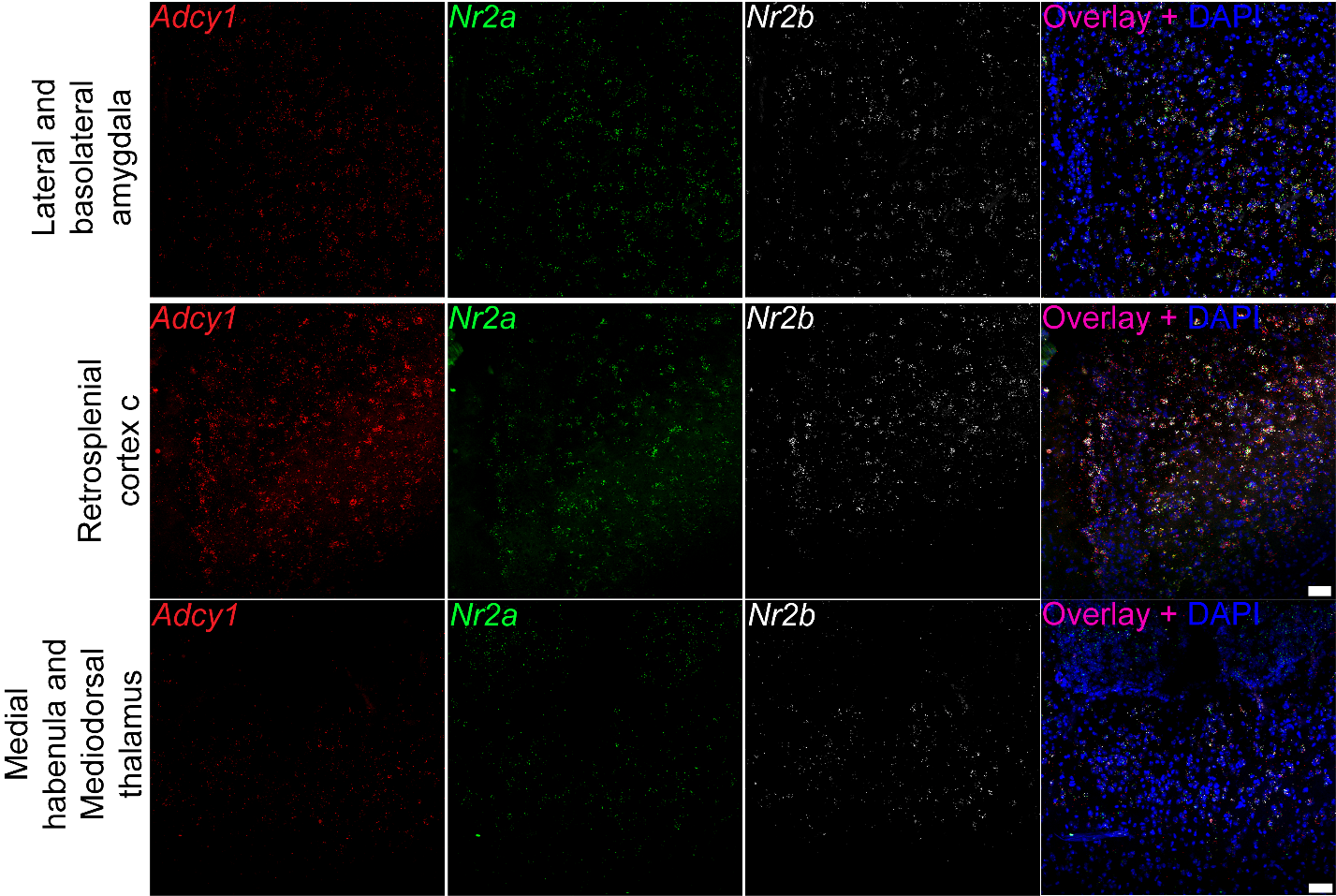


**Supplementary Figure 1. *Adcy1*, *Nr2a*, and *Nr2b* in the lateral and basolateral amygdala, retrosplenial cortex c, medial habenula, mediodorsal thalamus.** 20x; Scale bar = 50 µm.


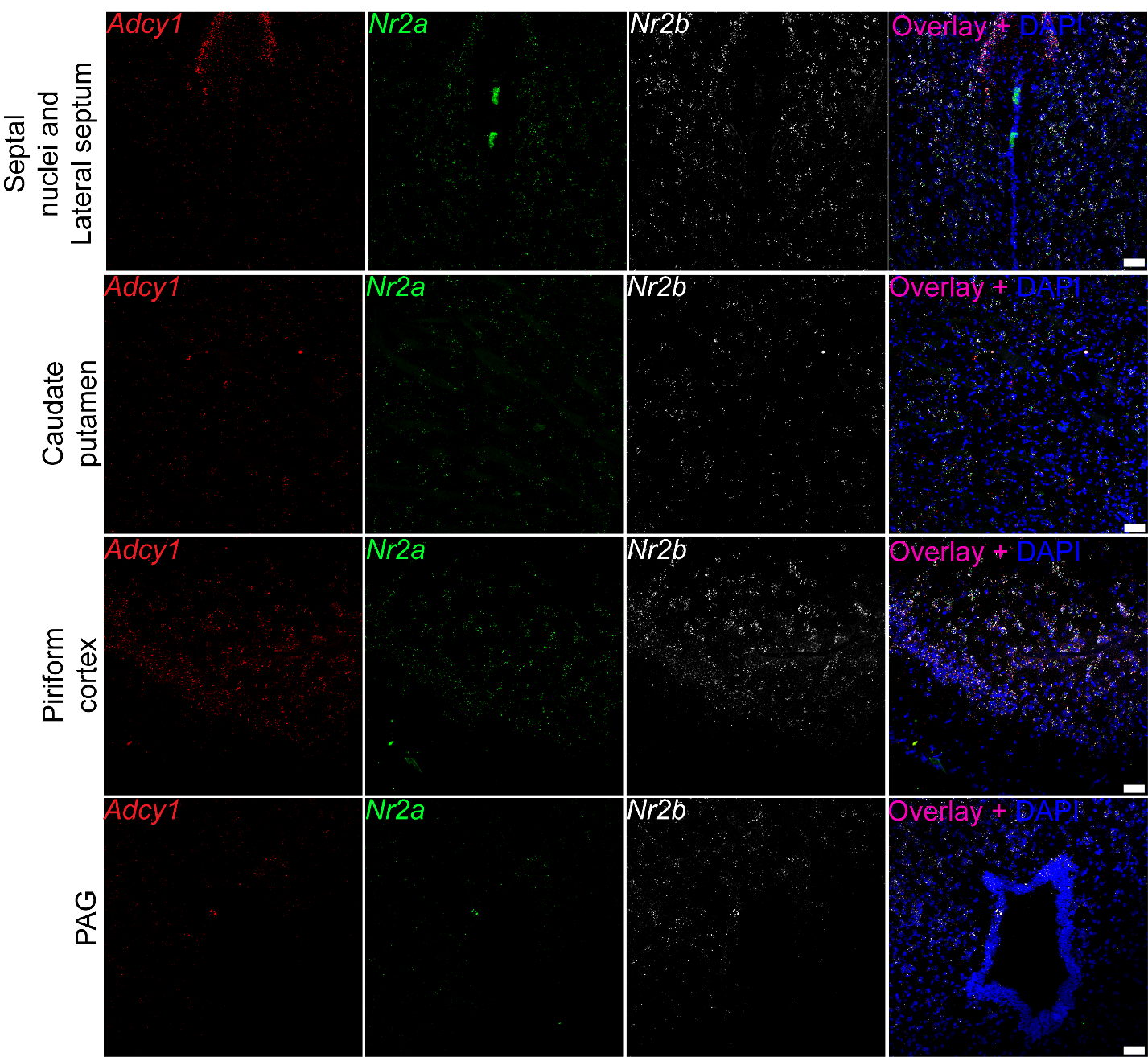


**Supplementary Figure 2. *Adcy1*, *Nr2a*, and *Nr2b* in the septal nuclei, lateral septum, caudate putamen, piriform cortex, periaqueductal gray (PAG), and spinal dorsal horn.** 20x; Scale bar = 50 µm.


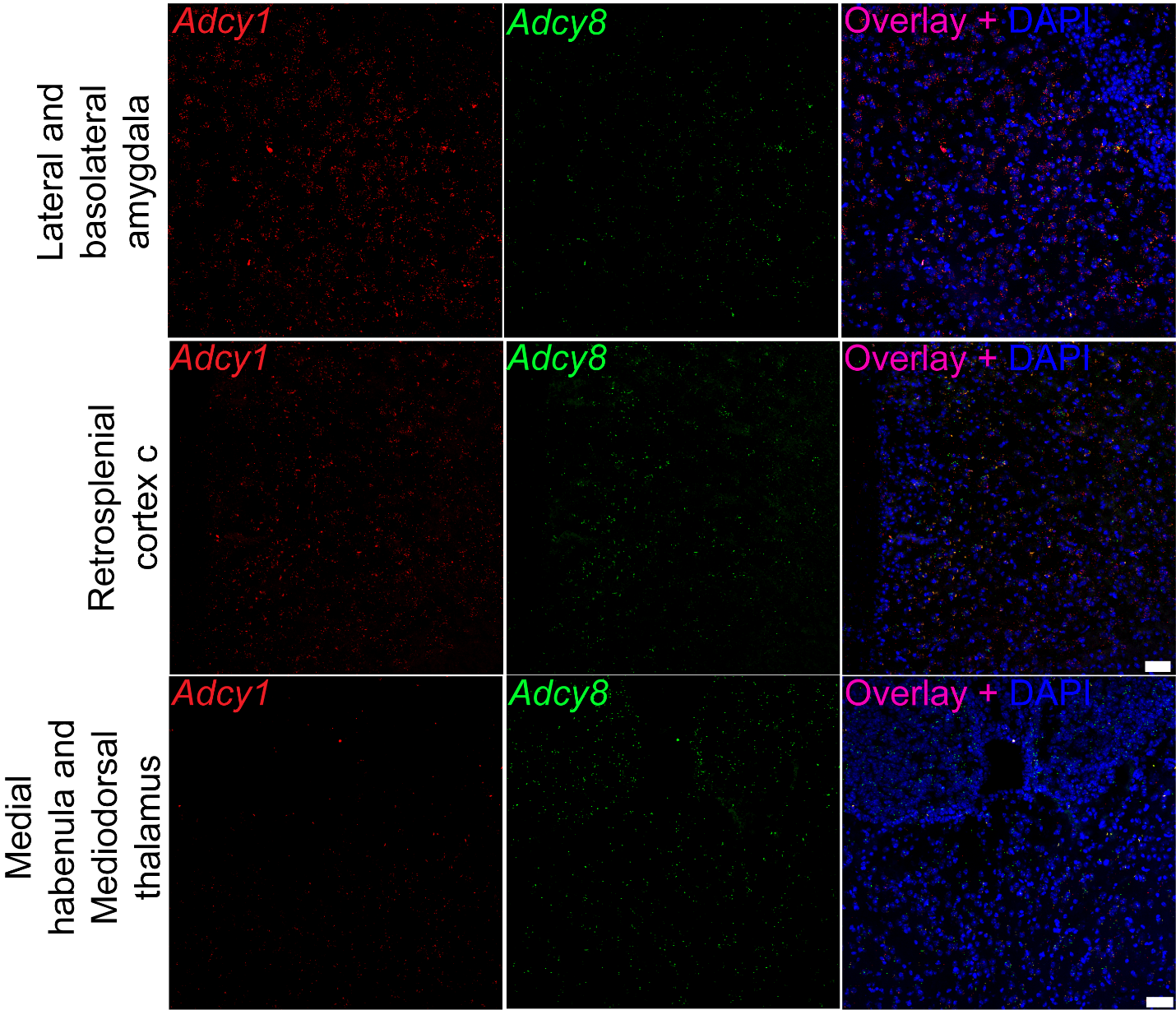


**Supplementary Figure 3. *Adcy1* and *Adcy8* in the lateral and basolateral amygdala, retrosplenial cortex c, medial habenula, mediodorsal thalamus.** 20x; Scale bar = 50 µm.


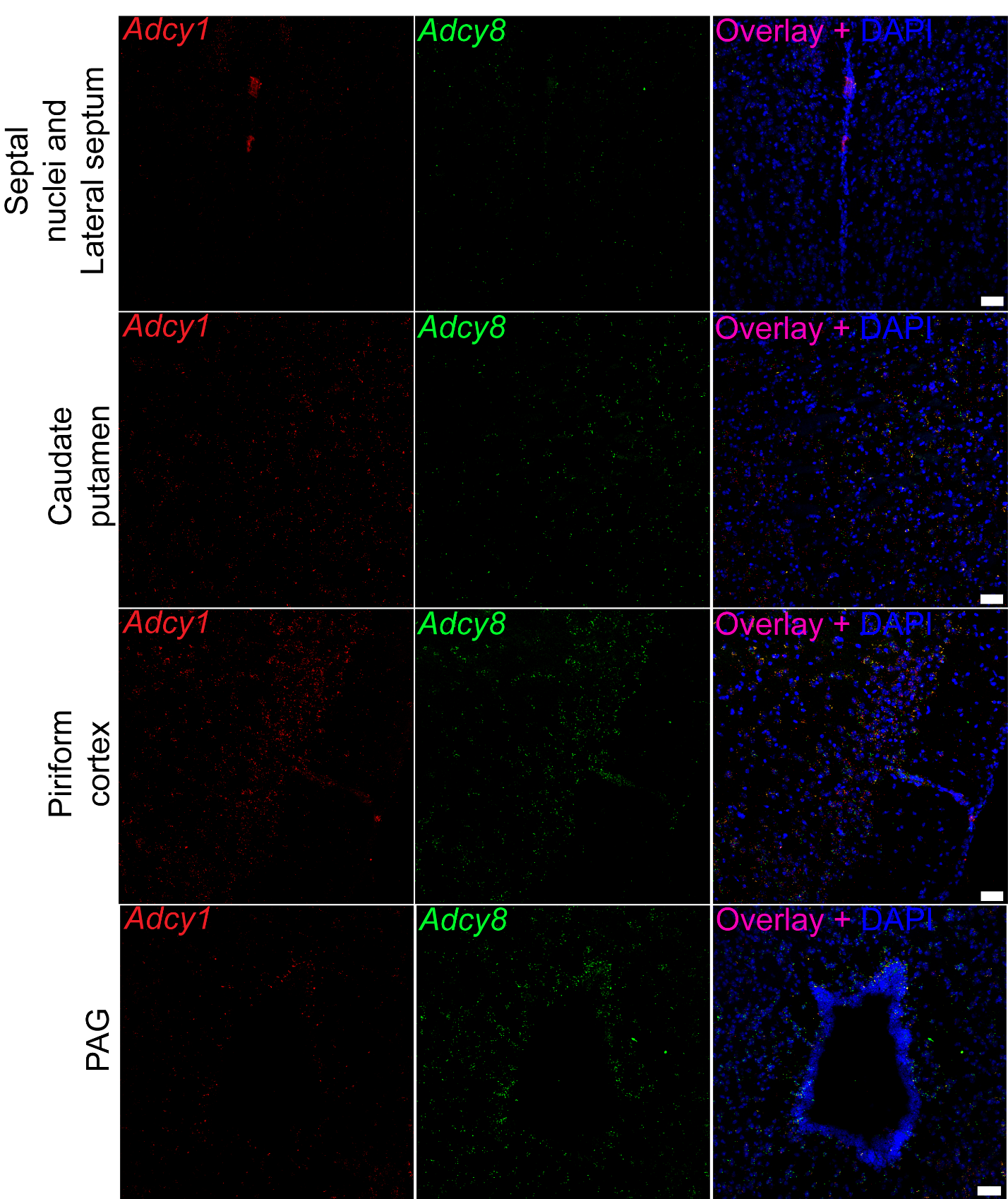


**Supplementary Figure 4. *Adcy1* and *Adcy8* in the septal nuclei, lateral septum, caudate putamen, piriform cortex, periaqueductal gray (PAG), and spinal dorsal horn.** 20x; Scale bar = 50 µm.


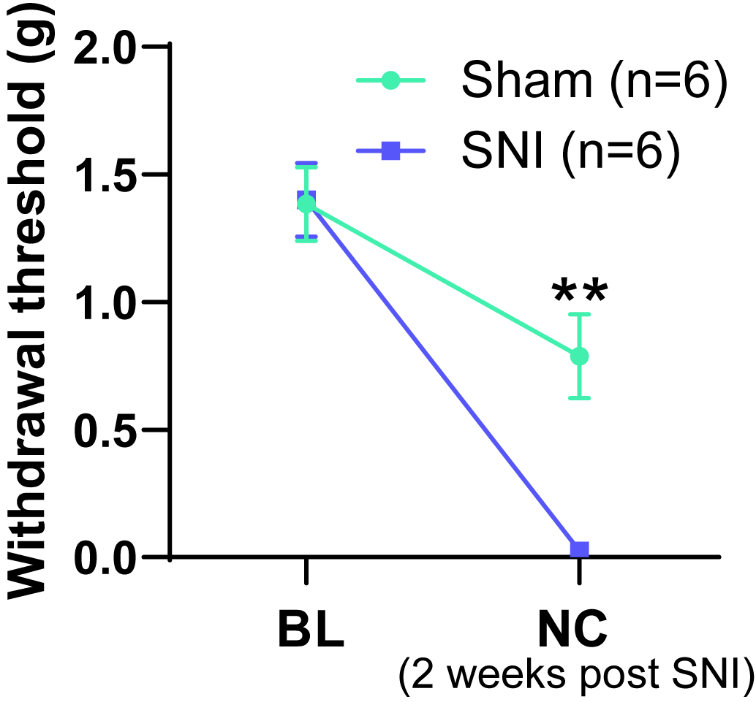


**Supplementary Figure 5. von Frey data for mice used in experiment 2 histology experiments.** Two-way ANOVA with Sidak multiple comparisons test **p<0.01
